# Revisiting the Relationship Between Systems of Innovation and Health Systems: A Response to Recent Commentaries

**DOI:** 10.15171/ijhpm.2019.85

**Published:** 2019-10-07

**Authors:** Pascale Lehoux, Federico Roncarolo, Hudson Silva, Antoine Boivin, Jean-Louis Denis, Rejean Hebert

**Affiliations:** ^1^Department of Health Management, Evaluation and Policy, School of Public Health, University of Montreal, Montreal, QC, Canada.; ^2^Institute of Public Health Research of University of Montreal (IRSPUM), University of Montreal, Montreal, QC, Canada.; ^3^Department of Family Medicine, University of Montreal, Montreal, QC, Canada.

We were very glad to read the six commentaries about our scoping review on the needs and challenges of health systems that responsible innovation in health (RIH) could address. The commentators offered great suggestions to further research on RIH and health systems. We provide clarifications to their queries and explain why revisiting the relationship between systems of innovation and health systems is necessary to realize a “giant leap” towards RIH.^1^

## Using the Human Development Index to Classify System-Level Needs and Challenges

While the reason we used the Human Development Index (HDI) to classify our data was unclear for van Olmen et al,^2^ Buttigieg^3^ underscored that RIH should be discussed in view of the values that underlie health systems. The assumption is that using a health system taxonomy would have showed variations that could be associated to different types of health system. The fact that only a small portion of the challenges reported in our corpus of articles fell into the “principles and values” (6%) category wherein the subcategory “inequalities” dominated by far lends support to the call for further research on how values such as solidarity, equity or universality get operationalized in health systems. The analytical thrust of our scoping review was not to account for what health systems “are and do,”^4^ but it would be enlightening to apply “causal loop diagramming”^2^ techniques to better address the interactions between health system components and innovation.

A classification based on the HDI has important strengths. First, it enabled us to include articles examining health systems in low- and middle-income countries that may not easily fit in taxonomies created for high-income countries. Second, by describing challenges reported in each of the four HDI groups, our findings captured the heterogeneity that exists within countries sharing a similar level of development. Third, the HDI combines indicators that are less volatile over time and directly relevant to population health.

## Differentiating Health SystemsFromSystems of Innovation

Peine^5^ and Stahl^6^ underscored the reciprocal relationship between health systems and innovations, a point we discussed elsewhere.^7^ What should have been understood as “two analytically separate entities”^5^ are not ‘health systems’ and ‘innovation.’ The distinction upon which RIH builds is between health systems and *systems* of innovation. Both follow from a set of institutionalized practices that have been shaped independently from one another to meet the aims of health policies, on the one hand, and of innovation policies, on the other hand. Though we agree that a dichotomy between ‘technology push’ and ‘demand pull’^4^ may be misleading, what RIH aims to achieve is to realign the goals pursued by systems of innovation with the goals of health systems. To move towards this aim, it is first necessary to articulate what health systems need and “want” from innovation.

Whereas Stahl^6^ found the aggregate data presented in our scoping review “too abstract,” Abrishami and Repping^1^ underscored the practical insights it offered to those who finance and develop innovations (eg, designing “readily-scalable solutions,” making entrepreneurs more “responsive” to public health priorities). If system-level demand remains unarticulated, innovators will pursue a technology-push strategy or turn to end-users for identifying needs and opportunities. While an individual-level approach is relevant for designing innovations that suit the needs of physicians, nurses, patients or informal caregivers, it cannot provide the systemic responsiveness that RIH requires. Responding to user demand is bound to reproduce innovations like the Da Vinci surgical robot described by van Lente.^4^

A “better diagnosis”^4^ of why health systems do not deliver their promises could start with recognizing that they were created decades ago and evolved along a path-dependent trajectory wherein innovations were geared at supporting medical practices and not at improving population health in an equitable and sustainable way. A focus on what is “medically required” left very little room to innovations providing a better response to chronic diseases, ageing and home care needs.

## Who Is Responsible for Responsible Innovation in Health?

It would seem presumptuous to disagree with the notion that steering technological development is very difficult.^2,4,6^ Yet, how do scholars explain the striking similarities between technologies developed in the past decades? Weren’t they developed with a certain demand in mind and a certain understanding of market opportunities? Today, we have the technologies that our systems of innovation were structured to generate and that our health systems were willing to pay for.^7^ What RIH suggests is that it is both possible and desirable to foreground system-level challenges as opportunities for innovation and to reward entrepreneurial activities that align with them.^8^

RIH calls for “an entire research programme”^6^ and Stahl^6^ nails down the key question: who is responsible for RIH? Our own work seeks to clarify the “overlapping networks”^6^ of responsibilities that connect stakeholders together. In a qualitative study, we found that Canadian health innovators were supportive of RIH and called for regulations and public policies to foster its implementation.^9^ In a meta-ethnography on why and how responsible innovations had been developed, we observed the key role of public policies, which can create transitory “protective niches” where novel kinds of innovation may be tested and adapted and where new players can enter the market and gain legitimacy.^10^ There are thus reasons to believe that many stakeholders feel responsible for RIH.

## Ethical issues

Not applicable.

## Competing interests

Authors declare that they have no competing interests.

## Authors’ contributions

All coauthor contributed to the manuscript and approved the final version.

## Authors’ affiliations

^1^Department of Health Management, Evaluation and Policy, School of Public Health, University of Montreal, Montreal, QC, Canada. ^2^Institute of Public Health Research of University of Montreal (IRSPUM), University of Montreal, Montreal, QC, Canada. ^3^Department of Family Medicine, University of Montreal, Montreal, QC, Canada.
